# Sweetened beverage consumption and the risk of hyperuricemia in Mexican adults: a cross-sectional study

**DOI:** 10.1186/1471-2458-14-445

**Published:** 2014-05-12

**Authors:** Joacim Meneses-Leon, Edgar Denova-Gutiérrez, Susana Castañón-Robles, Victor Granados-García, Juan O Talavera, Berenice Rivera-Paredez, Gerardo G Huitrón-Bravo, Margarita Cervantes-Rodríguez, Manuel Quiterio-Trenado, Samantha E Rudolph, Jorge Salmerón

**Affiliations:** 1 Unidad de Investigación Epidemiológica y en Servicios de Salud, Instituto Mexicano del Seguro Social, Cuernavaca, Morelos, México; 2 Centro de Investigación en Ciencias Médicas, Universidad Autónoma del Estado de México, Toluca, Estado de México, México; 3 Coordinación de Investigación en Salud, Centro Médico Nacional Siglo XXI, Instituto Mexicano del Seguro Social, México, D.F., México; 4 Unidad de Investigación en Economía de la Salud, Centro Médico Nacional Siglo XXI, Instituto Mexicano del Seguro Social, México, D.F., México; 5 Unidad de Investigación Médica en Epidemiologia Clínica, Centro Médico Nacional Siglo XXI, Instituto Mexicano del Seguro Social, México, D.F., México; 6 Facultad de Ciencias de la Salud, Universidad Autónoma de Tlaxcala, Tlaxcala, México; 7 Centro de Investigación en Salud Poblacional, Instituto Nacional de Salud Pública, Cuernavaca, Morelos, México; 8 UC Berkeley-UC San Francisco Joint Medical Program, Berkeley, CA, USA

**Keywords:** Sweetened beverages, Hyperuricemia, Mexican adults, Obesity

## Abstract

**Background:**

The prevalence of hyperuricemia has doubled worldwide during the last few decades. The substantial increase in sweetened beverage (SB) consumption has also coincided with the secular trend of hyperuricemia. Recent studies do show that the consumption of SB can induce hyperuricemia. However, the association between SB and hyperuricemia remains unclear. The aim of this study was to evaluate the association between SB consumption and levels of uric acid in Mexican adults.

**Methods:**

We performed a cross-sectional analysis of data from selected adults participating in the baseline assessment of the Health Workers Cohort Study. A total of 6,705 participants of both sexes between ages 18 and 70 years were included. SB intake was estimated using a validated semi-quantitative food frequency questionnaire. Biochemical and anthropometric information was collected using standard procedures. Hyperuricemia was defined as uric acid levels ≥ 7.0 mg/dL in men and ≥ 5.8 mg/dL in women. The association of interest was assessed by multiple logistic regression models.

**Results:**

The odds ratios (OR) for hyperuricemia in men who consume 0.5-1 SB/day was 1.59 (95% CI; 1.05-2.40) and 2.29 (95% CI; 1.55-3.38) for those who consume ≥3 SB/day when compared to men who consume less than half a SB/day. In women, the OR for hyperuricemia for those who consume >1.0- < 3.0 SB/day was 1.33 (95% CI; 1.04-1.70) and 1.35 (95% CI; 1.04-1.75) for those who consume ≥3 SB/day when compared to women who consume less than half a SB/day, independent of other covariables. Men and women with high SB consumption and a body mass index (BMI) ≥ 25 Kg/m^2^ had greater risk for hyperuricemia than men and women with low SB consumption and normal BMI < 25 Kg/m^2^.

**Conclusions:**

Our findings suggest that the consumption of SB is associated with an increased risk of hyperuricemia in Mexican adults. However, longitudinal research is needed to confirm the association between SB intake and hyperuricemia.

## Background

The prevalence of hyperuricemia, defined as increased serum uric acid levels [1], has doubled worldwide during the last few decades [2]. Hyperuricemia is the precursor of gout, which is the principal cause of inflammatory arthritis among adult men and older women [3]. Hyperuricemia is furthermore recognized as an independent risk factor for metabolic and cardiorenovascular conditions including metabolic syndrome, type 2 diabetes (T2DM) and insulin resistance [4-7]. In addition, epidemiological evidence suggests that hyperuricemia may play a role in epidemics of hypertension and coronary heart disease [8,9]; however, recent evidence suggests no causal association between uric acid and ischemic heart disease and blood pressure [10]. The worldwide population-level increase in serum uric acid levels over the past few decades correlates with pronounced changes in the most prevalent health risk factors known to be associated with hyperuricemina including: an increased prevalence of obesity and metabolic syndrome as well as changes in dietary patterns [11]. Studies suggest that foods such as alcohol, meat and seafood play an important role in the development of hyperuricemia [12-14]. In general, a purine-rich diet has been long recognized as a risk factor for hyperuricemia. Since uric acid is the end product of purine degradation, gout patients are advised to avoid purine-rich foods [15]. In contrast, dairy products and foods rich in vitamin C have been shown to be protective against hyperuricemia [16]. Although it is believed that diet plays an important role in the development of hyperuricemia, the association between other specific dietary factors or foods and hyperuricemia remains unclear.

Recent studies do show that the consumption of sugar-sweetened beverages (SB) can induce hyperuricemia [1,17,18]. Although these beverages do not contain purines, they do contain large quantities of sweeteners including sucrose (composed of 50% glucose and 50% fructose), fructose and high fructose corn syrup (typically composed of 45% glucose and 55% fructose) [19]. Both fructose and high fructose corn syrup have been associated with increased serum uric acid levels [20,21], since uric acid production is upregulated by fructose, unlike glucose and other monosaccharides [21]. The potential mechanisms underlying the detrimental effects of sucrose, fructose and high fructose corn syrup are under debate. Some studies have suggested insulin resistance as one of the mechanisms involved in hyperuricemia [22,23]. One recent study suggests that long-term consumption of fructose and high fructose corn syrup could be a source of reactive dicarbonyls that increases risk of metabolic disorders including hyperuricemia [24].

Mexico has one of the highest rates of SB consumption in the world [4]. Reports indicate that SB consumption among Mexican adults has increased to 21% of total caloric consumption from 1996 to 2006 [25,26], meaning SB consumption is a significant component of the current Mexican diet. For these reasons, a better understanding of how fructose can affect plasma urate concentrations is important if effective dietary recommendations are to be formulated. Current recommendations include restriction of alcohol, meat and other purine-rich foods, but planning recommendations concerning SB should also be considered [27].

In this study we assess the potential health impact of SB consumption by examining the relationship between SB intake and uric acid levels in Mexican adults participating in the Health Workers Cohort Study. Because earlier evidence [28] has suggested that body mass index (BMI) may modify the effect of sweetened beverage on gout or hyperuricemia, we also examined this relationship stratifying by BMI and age.

## Methods

### Study population

A cross-sectional analysis was performed using data from adults participating in the baseline evaluation of the ¨Health Workers Cohort Study¨ (2004–2006). The methodology and participant characteristics have been detailed previously [29]. Of the 8,977 adults formally enrolled, for the present analysis we used data from 8,514 participants aged 18 to 70 years, of which the following were excluded: pregnant women (n = 186), those with a diagnosis of gout (n = 67), those taking medications to control uric acid (n = 20), those taking diuretics (n = 79), those without uric acid values (n = 693), subjects that responded to less than 85% of questions or did not complete all the diet related questions (n = 525), subjects with extreme values of caloric intake (<600, > 7000 Kcal/day) (n = 87) using the standard deviation method (>3.86SD) [30], subjects with outlying values of energy consumption from SB (n = 74), subjects not providing information on tobacco use (n = 74) and subjects with incomplete anthropometric data (n = 4). Ultimately, a total of 6,705 subjects were included in the analysis.

This study was planned and performed according to the guidelines of the Declaration of Helsinki. All participating institutions’ research ethics committees [Comité de Ética en Investigación, Instituto Mexicano del Seguro Social (No. 12CEI 09 006 14); Comité de Ética en Investigación, Instituto Nacional de Salud Pública (No. 13CEI 17 007 36); Comité de Ética, Centro de Investigación en Ciencias Médicas (No. 1233008X0236)], approved the study protocol and informed consent forms. Written informed consent was obtained from all participants.

### Assessment of sugar- and artificially-sweetened beverage consumption and other dietary intake

To assess diet, a validated semi-quantitative food frequency questionnaire (FFQ) was used [31]. To test the FFQ’s reproducibility, it was administered twice, at a 1-year interval, to 134 women residing in Mexico city and the validity results were then compared with those from the set of 4 recall tests given at 3 month intervals. The questionnaire includes data describing the frequency of consumption of 116 foods during the previous year. This FFQ collects information about the consumption of soft drinks, such as: sugar-sweetened soft drinks (colas and other carbonated beverages with sugar), flavored water with sugar (such as lemon or orange water prepared with artificial flavorings) and diet soft drinks (low-calorie colas and other low-calorie beverages), using a standard serving size of 355 ml. We also assessed consumption of orange juice. The amount of energy intake from the ingestion of these beverages (Kcal/serving) was determined using the Evaluation System of Eating Habits and Nutrient Consumption (SNUT) [32]. For this study, we summed the intakes of single items to create totals for SB (sugar sweetened soft drinks and flavored water with sugar) and diet beverages (low-calorie colas and other low-calorie beverages). In addition, SB intake was divided into four categories: (I) < 0.5 servings of SB/day, (II) ≥0.5 – 1.0 servings of SB/day, (III) >1.0 - < 3 servings of SB/day, and (IV) ≥ 3 servings of SB/day. Total energy, energy from other carbohydrates, nutrient intakes and alcohol consumption were similarly estimated with this questionnaire.

### Biological measures

A fasting venous blood sample was collected from the antecubital vein; fasting time was ≥8 hours. Serum uric acid levels were determined with the enzymatic colorimetric method using the SYNCHRON CX® system [33]. Hyperuricemia was defined as uric acid levels ≥ 7.0 mg/dL in men and ≥ 5.8 mg/dL in women [1].

### Anthropometric measures

Body weight was measured with a previously calibrated electronic scale (model BC-533; Tanita, Tokyo, Japan), with participants wearing minimal clothing. Height was measured using a conventional stadiometer (SECA brand), on barefoot subjects standing with their shoulders in a normal position; measurements were taken with the tape in a horizontal plane perpendicular to the vertical scale, touching the top of the head at the moment of inspiration. Waist circumference was measured at the high point of the iliac crest at the end of normal expiration, to the nearest 0.1 cm, with a conventional steel measuring tape (SECA brand). Abdominal obesity was defined as a waist circumference > 102 cm for men and > 88 cm for women [34]. Body mass index was classified as: normal (18.5 to < 25 kg/m^2^), overweight (≥25 to < 30 kg/m^2^), or obese (≥30 kg/m^2^) [35]. The proportion of body fat was determined by dual X-ray absorptiometry (DEXA) using a Lunar densitometer (model: DPX-GE 73735, serial number: 638405U77) (Lunar Radiation Corporation, Madison, WI, USA; software version .35, fast scan mode). Excess body fat was defined as >35% in women and >25% in men [36]. All measurement procedures were performed by trained personnel using standardized procedures.

### Assessment of other variables

Demographic information was collected using a self-administered questionnaire. Physical activity level was determined using a survey used in similar follow-up studies [37,38]. The participants reported the time they spent each week on activities such as running, walking, etc. during a typical week in the previous year. The physical activity variable was categorized into: inactive <30 min/day and active ≥30 min/day, in accordance with previous studies [39]. Finally, participants were asked about weight changes they experienced during the previous year, and this information was categorized as: no weight change, weight loss, or weight gain in the past year. Participants who gained or lost less than 5 kg were categorized have experienced no weight change.

### Statistical analysis

We performed an exploratory analysis by sex and SB intake considering the variables of age, body mass index, body fat proportion, tobacco use, physical activity, levels of uric acid, SB consumption and other dietary variables. We used Student t tests for continuous variables and Chi^2^ tests for categorical variables to test for differences between sexes. When continuous variables did not show a normal distribution, non-parametric tests for trends across ordered groups were performed.

We assessed trends of acid uric levels, hyperuricemia, and other variables across categories of SB intake. Differences between groups were computed with the test for linear trend.

The effect of SB consumption on uric acid levels was determined using linear regression models. In this case, the increments of uric acid levels were considered across categories of SB intake, using the lowest category of consumption (<0.5 SB/day) as a reference.

To estimate the association between SB intake and levels of uric acid, we computed odds ratios (OR) and 95% confidence intervals (CI 95%) using multiple logistic regression models, adjusting for different covariables in several models. The first model was adjusted for age, the second added tobacco use, physical activity (min/day), and body weight changes, the third model added energy intake (kcal/day), alcohol consumption (g/day), percentage of energy from carbohydrates (quintiles), total meats (servings/day), seafood (servings/day), total fruits (servings/day), dairy products (servings/day), coffee (cups/day), and vitamin C intake (mg/day), and finally the fourth model added BMI (kg/m^2^) to determine whether or not the relationship was the result of a BMI classified as overweight or obese. In all the multivariate models, the reference category of energy intake from SB was defined as < 0.5 SB/day.

To assess possible effect modification, we explored analyses stratified by age (two categories: < 50 years vs. ≥ 50 years), and BMI (two categories: <25 kg/m^2^ vs. ≥25 kg/m^2^). We tested the significance of the interaction with a likelihood ratio test by comparing a model with the main effects of each intake and the stratifying variable and the interaction terms with a reduced model with only the main effects. All *p* values presented are two sided; *p* < 0.05 was considered statistically significant. All the statistical analyses were performed using the STATA statistical software package, version 9.2 for Windows (Stata Corp. LP: College Station, TX).

## Results

The study population was made up of mainly middle-aged participants; most (70.8%) were women with a mean age of 43 years, and the rest were men with a mean age of 42.6 years. Their average uric acid level was 5.9 mg/dL in men and 4.4 mg/dL in women (p < 0.001). A total of 20.6% of men had hyperuricemia as compared to 13.5% of women (p < 0.001). Men consumed an average of 225 kcal/day from SB, whereas women consumed 186 Kcal/day (p < 0.001) (Table 1).

**Table 1:** Characteristics of Mexican adults from the baseline assessment of the Health Workers Cohort Study

	**Men**	**Women**	
	**n = 1956 (29.2%)**	**n = 4749 (70.8%)**	*P*-value*
**Age (years)**^**Ş**^	42.6 (11.6)	43.0 (12.2)	0.1
18-27%	9.1	11.9	< 0.001
28-37	27.6	23.2	<0.001
38-47	29.4	29.4	0.9
48-57	22.3	22.3	0.9
58-70	11.6	13.2	0.1
**BMI (kg/m**^**2**^**)**^**Ş**^	27.1 (3.9)	26.7 (4.7)	< 0.001
Overweight % Ω	50.6	38.9	< 0.001
Obese % Ω	19.1	20.4	0.2
**Waist circumference (cm)**^**Ş**^	93.7 (10.9)	89.7 (12.7)	< 0.001
Central obesity % ¶	20.1	53.0	< 0.001
**Body fat proportion by mass**^**Ş**^	30.3 (7.3)	42.6 (6.6)	< 0.001
Excess (>25 M, >35 W), %	80.7	87.1	< 0.001
**Physical activity (min/d)**^**Ş**^	33.3 (39.0)	22.5 (28.5)	< 0.001
Active (≥30 min/d), %	41.0	31.8	< 0.001
**Tobacco use %**			
Ex-smoker	37.9	20.6	< 0.001
Current smoker	26.7	17.6	< 0.001
**Uric acid (mg/dL)**^**Ş**^	5.9 (1.5)	4.4 (1.2)	< 0.001
Hyperuricemia % **+**	20.6	13.5	< 0.001
**Dietary variables**			
Portions of sugar-sweetened beverages/day^**Ş**^	1.8 (1.6)	1.6 (1.5)	< 0.001
Portions of diet soft drinks	0.05 (0.2)	0.07 (0.3)	0.019
Portions of orange juice	0.36 (0.6)	0.42 (0.6)	<0.001
Calorie intake from sweetened beverages (Kcal/day)^**Ş**^	225 (198)	186 (184)	< 0.001
Percent of energy intake from sweetened beverages^**Ş**^	9.5 (7.2)	8.5 (7.2)	< 0.001
Total calorie intake (Kcal/day)^**Ş**^	2318 (925)	2146 (898)	< 0.001
Carbohydrates (% of energy)^**Ş**^	59.0 (8.5)	61.1 (8.6)	< 0.001
Fat (% of energy)^**Ş**^	20.3 (5.1)	20.5 (5.4)	0.2
Alcohol intake (gr/day)	9.2 (22.2)	2.2 (5.4)	< 0.001
Total meat intake (servings/day)^*a*^	1.5 (1.0)	1.3 (0.9)	< 0.001
Seafood intake (servings/day)^*b*^	0.15 (0.2)	0.14 (0.2)	0.05
Low fat dairy intake (serving/day)^*c*^	0.48 (0.9)	0.56 (1.0)	0.002
High fat dairy intake (servings/day)^*d*^	1.8 (1.6)	2.2 (2.1)	< 0.001
Coffee intake (servings/day)	0.5 (0.9)	0.5 (0.9)	0.8
Total fruit intake (servings/day)^*e*^	3.7 (3.5)	5.1 (5.0)	<0.001
Total vitamin C intake (mg/day)^*f*^	240.7 (206.6)	308.7 (261.8)	<0.001

^**Ş**^Values are means and with SD in parentheses, unless otherwise specified;

Ω BMI: Overweight ≥ 25 - < 30 kg/m^2^; Obese, when BMI ≥ 30 kg/m^2^;

¶ Central obesity: > 102 cm in men and > 88 cm in women;

**+** Hyperuricemia: ≥ 7.0 mg/dL for men, and ≥ 5.8 mg/dL for women;

^*a*^Total meat included main or mixed dish of beef/pork/lamb/, processed meat (sausage/bacon/ham/cecina/chorizo), poultry, chicken liver, beef liver, fried pork chunks;

^*b*^Seafood intake included tuna, sardines, other seafood;

^*c*^Low fat dairy foods included skim/low fat milk and panela cheese;

^*d*^High fat dairy foods included whole milk, cream, butter, ice cream, cream cheese, and other cheese.

^*e*^Total fruit intake included banana, plum, peach, apple, orange, grapes, strawberry, blackberry, melon, watermelon, mango, tangerine, pear, mammee apple, sapote, prickly pear, papaya, pineapple;

^*f*^Total vitamin C included dietary vitamin C and supplemental vitamin C;

*P < 0.05, difference in mean and proportion between sexes. Values were determined using a *student’s t-test* for continuous variable and a chi^2^ test for categorical variables.

Men with the highest SB intake (≥3 servings of SB/day) were younger, had higher caloric and total carbohydrate intakes and were more frequently smokers. Additionally, the male participants with the highest SB intake had higher average BMIs, proportions of obesity, and percentages of body fat than men in the category with the lowest SB intake (<0.5 servings of SB/day). Finally, serum uric acid concentrations and hyperuricemia prevalence were higher in men with the highest levels of SB intake (*p* for trend <0.001). For the female participants, women in the highest category of SB consumption were more frequently smokers and were less active than women in the lowest category of SB consumption (Table 2).

**Table 2:** Characteristics, according to categories of sugar-sweetened beverage intake by sex in a Mexican adult population

	**Men**					**Women**				
	**< 0.5 SB/day**	**≥ 0.5-1.0 SB/day**	**>1.0- < 3.0 SB/day**	**≥ 3 SB/day**		**< 0.5 SB/day**	**≥ 0.5-1.0 SB/day**	**>1.0- < 3.0 SB/day**	**≥ 3 SB/day**	
	**(n = 333)**	**(n = 425)**	**(n = 592)**	**(n = 606)**	***P value*** *	**(n = 1178)**	**(n = 1063)**	**(n = 1312)**	**(n = 1196)**	***P value*** *
**Age (years)**^**Ş**^	45.5 (12.0)	43.9 (11.8)	41.9 (11.4)	40.7 (11.0)	< 0.001	45.8 (12.7)	43.3 (12.3)	42.2 (11.7)	41.0 (11.4)	< 0.001
18-27%	5.0	7.9	10.4	10.8	0.003	9.5	12.2	12.7	13.2	0.004
28-37	22.3	24.8	28.7	31.4	0.003	19.5	22.6	23.6	27.1	< 0.001
38-47	28.2	30.2	28.9	30.0	0.6	25.8	28.8	31.0	31.7	0.001
48-57	27.3	22.4	22.1	19.8	0.01	24.4	22.9	22.5	19.3	0.003
58-70	17.2	14.7	9.9	8.0	< 0.001	20.8	13.5	10.2	8.7	<0.001
**BMI (kg/m**^**2**^**)**^**Ş**^	26.7 (3.8)	26.9 (4.0)	27.2 (4.0)	27.4 (3.8)	0.01	26.8 (4.8)	26.9 (4.7)	26.5 (4.6)	26.4 (4.7)	0.1
Overweight % Ω	50.5	52.8	49.1	50.6	0.9	39.4	37.9	38.1	40.3	0.6
Obese %	15.4	15.2	21.3	21.8	0.02	21.1	22.6	19.8	18.4	0.1
**Body fat proportion**	29.3 (6.9)	30.0 (7.6)	30.7 (7.8)	30.5 (6.7)	0.01	42.4 (6.7)	42.9 (6.5)	42.5 (6.7)	42.6 (6.5)	0.5
Excess body fat % ¶	75.8	78.7	82.2	83.2	0.01	86.1	88.2	86.7	87.6	0.3
**Physical activity (min/d)**^**Ş**^	33.8 (37.5)	31.0 (37.7)	33.2 (39.2)	34.8 (40.6)	0.7	25.2 (30.0)	22.9 (29.9)	19.5 (25.7)	22.7 (28.4)	0.05
Active (≥30 min/d) %	42.9	37.6	40.3	43.1	0.9	35.2	32.0	27.9	32.6	0.2
**Tobacco use** %										
Ex-smoker	40.1	42.1	37.3	34.4	0.1	22.1	19.4	19.8	21.0	0.5
Current smoker	16.3	20.1	30.6	33.2	< 0.001	13.2	14.8	20.4	21.1	< 0.001
**Uric acid (mg/dL)**^**Ş**^	5.7 (1.3)	5.8 (1.6)	5.9 (1.4)	6.1 (1.5)	< 0 .001	4.3 (1.3)	4.4 (1.2)	4.4 (1.2)	4.4 (1.3)	0.9
Hyperuricemia %+	13.4	19.2	20.8	25.2	< 0.001	13.1	13.8	14.1	14.1	0.9
**Dietary variables**										
Portion of sweetened beverages/day^**Ş**^	0.3 (0.4)	0.8 (0.4)	1.5 (0.7)	3.5 (1.6)	< 0.001	0.3 (0.4)	0.8 (0.4)	1.5 (0.8)	3.5 (1.5)	< 0.001
Calorie intake from sweetened beverages (kcal/day)^**Ş**^	34.9 (26.8)	102.2 (41.8)	196.2 (89.4)	442.4 (195.9)	< 0.001	28.9 (25.1)	96.8 (40.4)	180.2 (90.8)	426.4 (182.4)	< 0.001
Total calorie intake (kcal/day)^**Ş**^	2214 (1004)	2250 (827)	2324 (929)	2416 (931)	0.002	2035 (934)	2155 (840)	2111 (916)	2309 (871)	< 0.001
Carbohydrates (% of energy)^**Ş**^	56.9 (10.0)	57.2 (8.1)	57.8 (7.7)	62.7 (7.3)	< 0.001	59.5 (10.0)	59.4 (8.0)	60.2 (7.6)	65.1 (7.4)	< 0.001
Protein (% of energy)^**Ş**^	17.9 (7.7)	17.7 (6.0)	17.8 (6.0)	15.6 (4.9)	<0.001	15.0 (5.6)	15.2 (4.8)	15.2 (4.6)	13.0 (3.9)	< 0.001
Fat (% of energy)^**Ş**^	25.2 (6.1)	25.1 (5.3)	24.4 (4.9)	21.7 (4.6)	< 0 .001	24.5 (6.5)	25.4 (5.2)	24.5 (4.8)	21.8 (4.9)	< 0.001
Alcohol intake (gr/day)	10.2 (40.9)	9.3 (17.1)	10.0 (17.7)	7.7 (12.6)	0.18	2.1 (5.2)	2.5 (6.3)	2.0 (5.4)	2.1 (4.8)	1.00
Total meat intake (servings/day)^*a*^	1.45 (1.3)	1.47 (0.9)	1.54 (0.9)	1.46 (0.9)	0.88	1.19 (1.0)	1.33 (0.9)	1.32 (0.9)	1.26 (0–8)	0.06
Seafood intake (servings/day)^*b*^	0.20 (0.33)	0.15 (0.17)	0.13 (0.15)	0.13 (0.18)	< 0.001	0.15 (0.25)	0.15 (0.19)	0.13 (0.22)	0.12 (0.30)	0.08
Low fat dairy intake (serving/day)^*c*^	0.62 (1.0)	0.53 (1.0)	0.46 (0.8)	0.39 (0.8)	< 0.001	0.70 (1.1)	0.60 (1.0)	0.48 (0.9)	0.47 (0.9)	<0.001
High fat dairy intake (servings/day)^*d*^	1.8 (1.8)	1.9 (1.8)	1.7 (1.4)	1.7 (1.5)	0.36	2.3 (2.4)	2.3 (2.0)	2.1 (1.9)	2.0 (1.9)	0.003
Coffee intake (servings/day)	0.44 (0.9)	0.47 (0.9)	0.54 (0.9)	0.55 (0.9)	0.05	0.44 (0.8)	0.46 (0.8)	0.58 (0.9)	0.57 (0.9)	<0.001
Total fruit intake (servings/day)^*e*^	4.8 (5.2)	3.9 (4.2)	3.6 (3.9)	3.1 (3.5)	<0.001	6.0 (6.4)	5.4 (5.3)	4.7 (5.0)	4.5 (4.5)	< 0.001
Total vitamin C intake (mg/day)^*f*^	278 (256)	237 (185)	230 (199)	230 (196)	0.001	330 (298)	303 (239)	287 (256)	317 (247)	0.24

^**Ş**^Values are means and with SD in parentheses, unless otherwise specified;

Ω BMI: Overweight ≥ 25 - < 30 kg/m^2^; Obese, when BMI ≥ 30 kg/m^2^;

¶ Central obesity: > 102 cm in men and > 88 cm in women;

**+** Hyperuricemia: ≥ 7.0 mg/dL for men, and ≥ 5.8 mg/dL for women;

^*a*^Total meat included main or mixed dish of beef/pork/lamb/, processed meat (sausage/bacon/ham/cecina/chorizo), poultry, chicken liver, beef liver, fried pork chunks;

^*b*^Seafood intake included tuna, sardines, other seafood;

^*c*^Low fat dairy foods included skim/low fat milk and panela cheese;

^*d*^High fat dairy foods included whole milk, cream, butter, ice cream, cream cheese, and other cheese.

^*e*^Total fruit intake included banana, plum, peach, apple, orange, grapes, strawberry, blackberry, melon, watermelon, mango, tangerine, pear, mammee apple, sapote, prickly pear, papaya, pineapple;

^*f*^Total vitamin C included dietary vitamin C and supplemental vitamin C;

*P-values were determined using a test for linear trend for continuous variables and chi^2^ test for categorical variables.

The results of a multivariate regression analysis evaluating the effect of sweetened beverage intake on uric acid levels are presented in Table 3. In the adjusted model, we found that uric acid levels in men who consumed more than 3 servings of sweetened beverages per day increased 0.38 mg/dL (*P* < 0.001); whereas in women these levels increased to 0.21 mg/dL, in comparison with subjects who consumed less than 0.5 servings of SB/day.

**Table 3:** Differences in serum uric acid levels (mg/dL) according to categories of sugar-sweetened beverage intake

	**Men**					Women**				
	**< 0.5 SB/day**	**≥ 0.5-1.0 SB/day**	**>1.0- < 3.0 SB/day**	**≥ 3 SB/day**		**< 0.5 SB/day**	**≥ 0.5-1.0 SB/day**	**>1.0- < 3.0 SB/day**	**≥ 3 SB/day**	
	**β (95% CI)**	**β (95% CI)**	**β (95% CI)**	**β (95% CI)**	P-value*	**β (95% CI)**	**β (95% CI)**	**β (95% CI)**	**β (95% CI)**	P-value*
Model I	0.0	0.16 (−0.04,0.38)	0.24 (0.04,0.44)	0.45 (0.26,0.65)	<0.001	0.0	0.15 (0.05,0.25)	0.19 (0.10,0.28)	0.14 (0.05,0.24)	0.002
Model II	0.0	0.16 (−0.05,0.37)	0.22 (0.02,0.42)	0.42 (0.22,0.62)	<0.001	0.0	0.15 (0.05,0.25)	0.14 (0.04,0.23)	0.18 (0.08,0.27)	0.004
Model III	0.0	0.15 (−0.05,0.36)	0.18 (0.01,0.38)	0.42 (0.22,0.62)	<0.001	0.0	0.15 (0.5,0.24)	0.17 (0.07,0.26)	0.20 (0.10,0.29)	<0.001
Model IV	0.0	0.14 (−0.06,0.35)	0.15 (−0.04,0.35)	0.38 (0.18,0.57)	<0.001	0.0	0.14 (0.04,0.23)	0.18 (0.09,0.27)	0.21 (0.11,0.30)	<0.001

*P for linear trend.

Model I: adjusted for age (18–27, 28–37, 38–47, 48–57, 58–70 years of age);

Model II: adjusted for tobacco use (Current smoker, ex-smoker y non-smoker), physical activity (active ≥30 min/d, inactive < 30 min/d), weight change (did not gain, gained, lost);

Model III: adjusted for calorie intake (quintiles),total meats (servings/day), seafood (servings/day), dairy products (servings/day), alcohol intake (g/day), total fruits (servings/day), vitamin C intake (mg/day), coffee (cups/day);

Model IV: BMI (normal <25 kg/m^2^, overweight/obesity ≥25 kg/m^2^);

**For women, model IV was additionally adjusted for menopause (%) and postmenopausal hormone use (%).

Results of the multiple logistic regression analysis show a positive association between SB consumption and the presence of hyperuricemia in men and women. In men consuming ≥0.5 - 1.0 servings of SB/day, the multivariate odds ratio for hyperuricemia was 1.59 (95% CI: 1.05–2.40), as compared with the reference consumption level of SB (<0.5 servings of SB/day). For men consuming >1.0 - <3.0 servings of SB/day, this was 1.68 (95% CI: 1.14–2.49), and men consuming ≥ 3.0 SB/day was 2.29 (95% CI: 1.55–3.38; p for trend <0.001). For women, the ORs for hyperuricemia across categories of SB consumption were: 1.19 (95% CI: 0.92-1.53), 1.33 (95% CI: 1.04-1.70) and 1.35 (95% CI: 1.04-1.75) *p* for trend 0.015 (Table 4).

**Table 4:** Odds ratios (95% CI) for hyperuricemia according to categories of energy of sweetened beverage intake

	**Men**					Women**				
	**< 0.5 SB/day**	**≥ 0.5-1.0 SB/day**	**>1.0- < 3.0 SB/day**	**≥ 3 SB/day**		**< 0.5 SB/day**	**≥ 0.5-1.0 SB/day**	**>1.0- < 3.0 SB/day**	**≥ 3 SB/day**	
	**OR (95% CI)**	**OR (95% CI)**	**OR (95% CI)**	**OR (95% CI)**	***P-value*** *	**OR (95% CI)**	**OR (95% CI)**	**OR (95% CI)**	**OR (95% CI)**	***P-value*** *
Model I	1.0	1.53 (1.03-2.28)	1.73 (1.19-2.51)	2.22 (1.54-3.21)	<0.001	1.0	1.23 (0.96-1.58)	1.35 (1.06-1.71)	1.34 (1.04-1.71)	0.014
Model II	1.0	1.51 (1.01-2.24)	1.67 (1.14-2.43)	2.14 (1.47-3.10)	<0.001	1.0	1.22 (0.95-1.57)	1.32 (1.04-1.68)	1.33 (1.04-1.71)	0.016
Model III	1.0	1.62 (1.07-2.43)	1.76 (1.19-2.60)	2.40 (1.63-3.53)	<0.001	1.0	1.22 (0.95-1.56)	1.30 (1.02-1.66)	1.32 (1.02-1.71)	0.026
Model IV	1.0	1.59 (1.05-2.40)	1.68 (1.14-2.49)	2.29 (1.55-3.38)	<0.001	1.0	1.19 (0.92-1.53)	1.33 (1.04-1.70)	1.35 (1.04-1.75)	0.015

*P for linear trend.

Model I: adjusted for age (18–27, 28–37, 38–47, 48–57, 58–70 years of age);

Model II: adjusted for tobacco use (Current smoker, ex-smoker y non-smoker), physical activity (active ≥30 min/d, inactive < 30 min/d), weight change (did not gain, gained, lost);

Model III: adjusted for calorie intake (quintiles),total meats (servings/day), seafood (servings/day), dairy products (servings/day), alcohol intake (g/d), total fruits (servings/day), vitamin C intake (mg/d), coffee (cups/day);

Model IV: BMI (normal <25 kg/m^2^, overweight/obesity ≥25 kg/m^2^);

**For women, model IV was additionally adjusted for menopause (%) and postmenopausal hormone use (%).

**Figure 1:**
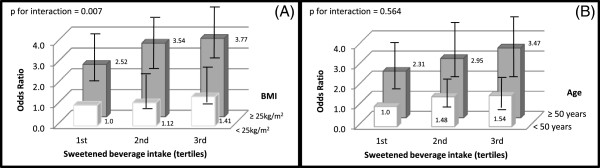
**Multivariate odds ratio of hyperuricemia in men, according to the tertiles of sweetened beverage intake.** *Analyses of effect modification: **(A)** stratified by body mass index (BMI: < 25 kg/m2 vs ≥ 25 kg/m2), **(B)** stratified by age (< 50 years vs ≥ 50 years. Reference group for comparisons was men in lowest tertile of sweetened beverage intake and **(A)** with body mass index < 25 kg/m2, and **(B)** with age < 50 years. Odds ratio were adjusted for: age (years), tobacco use (current smoker, ex-smoker y non-smoker), physical activity (active ≥ 30 min/day, inactive < 30 min/day), weight change (did not gain, gained, lost), calorie intake (quintiles), percentage of energy from total carbohydrate (quintiles), percentage of energy from protein (quintiles), alcohol intake (gr/day), vitamin C intake (mg/day), and body mass index.

**Figure 2:**
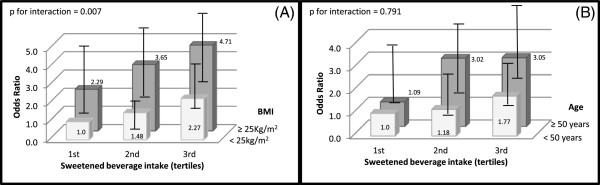
**Multivariate odds ratio of hyperuricemia in women, according to the tertiles of sweetened beverage intake.** *Analyses of effect modification: **(A)** stratified by body mass index (BMI: < 25 kg/m2 vs. ≥ 25 kg/m2), **(B)** stratified by age (< 50 years vs. ≥ 50 years. Reference group for comparisons was women in lowest tertile of sweetened beverage intake and **(A)** with body mass index < 25 kg/m2, and **(B)** with age < 50 years. Odds ratio were adjusted for: age (years), tobacco use (current smoker, ex-smoker y non-smoker), physical activity (active ≥ 30 min/d, inactive < 30 min/d), weight change (did not gain, gained, lost), calorie intake (quintiles), percentage of energy from total carbohydrate (quintiles), percentage of energy from protein (quintiles), alcohol intake (g/d), vitamin C intake (mg/d), and body mass index.

We examined the joint effect of SB intake and body mass index by cross classifying the study population by both variables. The odds ratio from these stratified analyses was 4.71 (95% CI: 2.64-8.36; *p* for interaction 0.007) when men with high SB consumption and overweight/obesity were compared with men with a low consumption of sweetened beverage and body mass index < 25 kg/m^2^ (Figure 1A). In women (Figure 2A), the OR for the combination of a high consumption of SB and BMI ≥ 25 kg/m^2^ compared with women in the opposite extreme was 3.77 (95% CI: 2.53-5.35, *p* for interaction 0.007).

## Discussion

Our results suggest that a considerable portion of our male participants’ energy consumption came from SB (9.5%). This result is consistent with previous reports that estimate the daily energy intake from SB among Mexican adults (10%) [4,26]. We found that men consumed an average of 225 Kcal/day and women consumed 186 Kcal/day from SB. These findings suggest that excessive SB consumption is contributing to excess calorie intake [25], supporting recommendations to minimize daily SB consumption. These recommendations are further supported given that our study population exceeded the recommended calorie intake limit (220 Kcal/day for men and 180 Kcal/day for women) established for the Mexican population by a committee of experts [26].

According to our results, increased SB intake is positively associated with increased risk of hyperuricemia in Mexican adults, independent of other covariables. The prevalence of hyperuricemia in our study (20.6% of men, 13.5% of women) is similar to that reported in a cross-sectional analysis completed by Choi et al. (19% of men, 17% of women) in the US using information from the Third US National Health and Nutrition Examination Survey, in which the association between SB consumption and levels of uric acid was analyzed [1].

In this study, Choi et al. reports that the subjects consuming more than 4 SB/day had an 82% increased risk of hyperuricemia (OR = 1.82; *p* = 0.003) in comparison with those that did not consume SB [1]. In our results, the risk of hyperuricemia increased significantly with an intake of SB greater than 3 SB/day, leading to 129% increased odds of hyperuricemia in men and 35% increased odds of hyperuricemia in women compared with participants who consumed less than 0.5 servings of SB/day.

In this study, we observed that men and women with high SB consumption and a body mass index ≥ 25 Kg/m^2^ are respectively at approximately 5.0 and 4.0 times greater risk for hyperuricemia than men and women with low SB consumption and normal body mass index, independent of dietary and other risk factors. Obesity is associated with hyperuricemia [40], and also positively and independently associated with clinical conditions commonly related to hyperuricemia including insulin resistance, hypertension, and inflammation [41].

The effect of SB consumption on uric acid levels may be explained by changes resulting from the ingestion of sucrose and fructose. During the metabolism of fructose, it is phosphorylated to fructose-1-phosphate (F1P) by fructokinase. The depletion of adenosine triphosphate (ATP) inhibits the oxidative phosphorylation of adenosine diphosphate (ADP) by creating a shortage of inorganic phosphate (P*i*). As a result, F1P is sequestered and the ADP produced during this metabolic cycle is converted into adenosine monophosphate (AMP) by adenylate kinase, which can also serve as a substrate for the production of uric acid. Moreover, the depletion of ATP and P*i* during the metabolism of fructose decreases feedback inhibition for the generation of uric acid. Indeed, experimental studies in humans and animals show a short term rise in acid uric concentrations with subsequent fructose ingestion or infusion [20,21]. Fructose may also indirectly increase serum levels of uric acid by increasing insulin resistance, leading to impaired glucose tolerance, and elevated circulating insulin levels [18,20,21,42,43].

Our findings have important implications for the prevention of hyperuricemia. Our results provide evidence that SB poses an increased risk for hyperuricemia. This casts doubt on the adequacy of the current dietary recommendations of the Mexican public health system aimed at reducing SB consumption. New health policies are necessary. While obesity is an important risk factor for T2DM, and other metabolic complications, there is new evidence suggesting that elevated uric acid levels is one of the most important risk factors for cardiovascular disease and also plays an important role in the development of renal disease [5], T2DM metabolic syndrome, and insulin resistance [44,45], which are important public health problems in Mexico.

There are some potential limitations of our study. First, the cross-sectional design of the study does not allow for identification of a causal relationship between SB intake and the occurrence of hyperuricemia, since the temporal relationship of these events cannot be determined. The relationship between high SB consumption and hyperuricemia is, however, biologically plausible, and will need to be confirmed in future longitudinal studies. Second, this cohort population cannot be considered representative of the Mexican population as a whole, but instead only representative of adults living in urban areas in central Mexico. Finally, even though many studies, including ours, used ≥ 7 mg/dL for men and ≥ 5.8 mg/dL for women as a cut-off to identify hyperuricemia, there is no consensus on the acid uric level cut-off point for identifying hyperuricemia. In spite of these limitations, the main strength of this study was the large number of participants.

## Conclusion

Our findings support a positive association between SB intake and serum uric acid levels in Mexican adults. If this relationship is causal, it could inform approaches for preventing chronic diseases such as T2DM, hypertension, gout, and cardiovascular disease. This evidence furthermore suggests the need to implement health policies for reducing SB consumption in Mexico. Longitudinal research is needed to confirm the association between SB intake and hyperuricemia. Further studies are also required to elucidate the risk of hyperuricemia among high SB consuming overweight/obese individuals and the elderly.

## Abbreviations

T2DM: Type 2 diabetes mellitus; SB: Sweetened beverages; BMI: Body mass index; FFQ: Food frequency questionnaire; SNUT: Evaluation system of eating habits and nutrient composition; DEXA: Dual X-ray absorptiometry; OR: Odds ratio; CI 95%: Confidence intervals; F1P: Fructose-1-phosphate; ATP: Adenosine triphosphate; ADP: Adenosine diphosphate; Pi: Inorganic phosphate; AMP: Adenosine monophosphate.

## Competing interest

We declare that there is no competing interest in submitting this manuscript for publication to BMC Public Health.

## Authors’ contributions

ED-G and JS designed the study; JM-L, ED-G, JS, SC-R, BR-P, GH-B, MQ-T, collected the data; JM-L, ED-G, JS performed the statistical analyses, JM-L, MC-R, SR, ED-G, JS, drafted the manuscript. All authors reviewed and commented on the manuscript. All authors read and approved the final version of the paper.

## Pre-publication history

The pre-publication history for this paper can be accessed here:

http://www.biomedcentral.com/1471-2458/14/445/prepub
